# Classification of occupational activity categories using accelerometry: NHANES 2003–2004

**DOI:** 10.1186/s12966-015-0235-z

**Published:** 2015-06-30

**Authors:** Jeremy A. Steeves, Catrine Tudor-Locke, Rachel A. Murphy, George A. King, Eugene C. Fitzhugh, Tamara B. Harris

**Affiliations:** Department of Kinesiology, University of Wisconsin-Milwaukee, 2400 E Hartford Avenue, Milwaukee, WI 53201 USA; Walking Behavior Laboratory, Pennington Biomedical Research Center, Baton Rouge, LA USA; Laboratory of Epidemiology and Population Sciences, National Institute on Aging, Bethesda, MD USA; Department of Kinesiology, University of Texas, El Paso, TX USA; Department of Kinesiology, Recreation, and Sports Studies, University of Tennessee, Knoxville, TN USA

**Keywords:** Occupational activity, Accelerometer, Work, Employment, Classification, Cross-sectional

## Abstract

**Background:**

An individual’s occupational activity (OA) may contribute significantly to daily physical activity (PA) and sedentary behavior (SB). However, there is little consensus about which occupational categories involve high OA or low OA, and the majority of categories are unclassifiable with current methods. The purpose of this study was to present population estimates of accelerometer-derived PA and SB variables for adults (*n* = 1112, 20–60 years) working the 40 occupational categories collected during the 2003–2004 National Health and Nutrition Examination Survey (NHANES).

**Methods:**

ActiGraph accelerometer-derived total activity counts/day (TAC), activity counts/minute, and proportion of wear time spent in moderate-to-vigorous PA [MVPA], lifestyle, and light PA organized by occupational category were ranked in ascending order and SB was ranked in descending order. Summing the ranks of the six accelerometer-derived variables generated a summary score for each occupational category, which was re-ranked in ascending order. Higher rankings indicated higher levels of OA, lower rankings indicated lower levels of OA. Tertiles of the summary score were used to establish three mutually exclusive accelerometer-determined OA groupings: high OA, intermediate OA, and low OA.

**Results:**

According to their summary score, ‘farm and nursery workers’ were classified as high OA and ‘secretaries, stenographers, and typists’ were classified as low OA. Consistent with previous research, some low OA occupational categories (e.g., ‘engineers, architects, and scientists’, ‘technicians and related support occupations’, ‘management related occupations’, ‘executives, administrators, and managers’, ‘protective services’, and ‘writers, artists, entertainers, and athletes’) associated with higher education and income had relatively greater amounts of MVPA compared to other low OA occupational categories, likely due to the greater percentage of men in those occupations and/or the influence of higher levels of leisure time PA. Men had more TAC, activity counts/minute and time in MVPA, but similar proportions of SB compared to women in all three OA groupings.

**Conclusions:**

Objectively measured PA allowed for a more precise estimate of the amount of PA and SB associated with different occupations and facilitated systematic classification of the 40 different occupational categories into three distinct OA groupings. This information provides new opportunities to explore the relationship between OA and health outcomes.

## Background

The benefits of regular physical activity (PA), including lowered mortality rates, improved function and enhanced quality of life, are widely recognized [1–4]. An individual can accumulate daily PA through occupational demands, transportation, household tasks, or engagement in leisure time PA (LTPA) [5, 6]. Higher levels of LTPA have been promoted as health enhancing, [2, 3] while the effects of occupational activity (OA) on health remain inconclusive [7–9]. Many studies have shown high levels of OA to be associated with improved health, [7, 10–13] and the detrimental effects of large amounts of occupational sitting [14, 15]. In contrast, other studies have found high levels of OA to have deleterious health effects [9, 16–19].

Due to the substantial amount of time relegated to paid work in today’s society [20–22], an individual’s occupation likely has a strong influence on daily PA and sedentary behaviors (SB) (e.g., does their job require mostly sitting, standing, walking, engaging in repetitive tasks, or heavy labor) [23]. Also OA and LTPA patterns are profoundly different in varying occupations. Blue collar workers may be highly physically active during work, and highly sedentary during leisure, while white collar workers may engage in greater LTPA, after a sedentary day at the office [24, 25]. While extensive research on the comparison of physical demands between different occupations has been performed for decades [26–28], the incorporation of OA into the measurement of daily PA is still evolving [29, 30]. Including OA in addition to LTPA will allow a better understanding of the prevalence of daily PA, and inform interventions aimed to improve health, increase productivity, and reduce work-related injury of the employed population.

Based on self-reported occupation, the National Health and Nutrition Examination Survey (NHANES) III used the 1980 U.S. Census Bureau Classification Codes to classify respondents into 40 defined and diverse occupational categories according to work performed, required skills, education, training, and credentials [31] (Table 1). Previously King et al. [32] used the occupation descriptions of the U.S. Department of Labor as a reference to assign each of the 40 occupational categories to one of three broad OA groupings: (a) high amounts of OA, (b) low amounts of OA, or (c) unclassifiable amounts of OA [32]. Seven of the 40 occupational categories were considered to represent high amounts of OA (e.g., ‘cleaning and building service’, ‘construction laborers’) and 10 were considered to represent low amounts of OA (e.g., ‘secretaries’, ‘teachers’). The remaining 23 occupational categories were considered too ambiguous to classify as either high or low OA and thus were labeled as unclassifiable OA (e.g., ‘health services’, ‘sales workers, retail and personal services’) (Table 1). These three general groupings have been used previously by other researchers [12, 21, 33], with analyses focused primarily on the two more extreme and certain groupings. Since the unclassifiable OA group contains the majority of the occupational categories, (23 of the 40 categories) limiting analysis to only the high and low OA groupings restricts what can be inferred about the relationship between OA and health outcomes.

**Table 1:** High, low, and unclassifiable occupational activity (OA) groupings assigned by an expert panel

Occupational categories previously classified as having high OA (*n* = 7)
Waiters and waitresses
Cleaning and building service
Farm and nursery workers
Construction trades
Construction laborers
Laborers, except construction
Freight, stock, and material movers (hand)
Occupational categories previously classified as having low OA (*n* = 10)
Executive, administrators, and managers
Management related
Engineers, architects and scientists
Teachers
Secretaries, stenographers, and typists
Information clerks
Records processing
Material recording, scheduling, and distributing clerks
Miscellaneous administrative support
Motor vehicle operators
Occupational categories previously classified as having unclassifiable OA (*n* = 23)
Health diagnosing, assessing and treating
Writers, artists, entertainers, and athletes
Other professional specialty
Technicians and related support
Supervisors and proprietors, sales
Sales representatives, finance, business, & commodities ex. Retail
Sales workers, retail and personal services
Private household
Protective service
Cooks
Miscellaneous food preparation and service
Health service
Personal service
Farm operators, managers, and supervisors
Related agricultural, forestry, and fishing
Vehicle and mobile equipment mechanics and repairers
Other mechanics and repairers
Extractive and precision production
Textile, apparel, and furnishings machine operators
Machine operators, assorted materials
Fabricators, assemblers, inspectors, and samplers
Other transportation and material moving
Other helpers, equipment cleaners, hand packagers and laborers

OA determined from the 1980 U.S. Census Bureau Classification Coding System

The 2003–2004 NHANES contained the same 40 occupational categories, and included objectively measured PA and SB collected with accelerometry. This serendipitous coupling presented an opportunity to categorize individuals into new OA groupings using objective data. Therefore, the purpose of this analysis of the 2003–2004 NHANES occupation and accelerometry data was to classify the 40 occupational categories into high OA, intermediate OA, and low OA groupings based on objectively measured PA and SB.

## Methods

As a continuous surveillance program conducted by the National Center for Health Statistics (NCHS), the NHANES assesses the health and nutritional status of non-institutionalized U.S. civilians [34]. Complex, multi-stage sampling was used to obtain a nationally representative sample. This analysis focused on a subgroup of the total population; specifically, employed individuals aged 20–60 years. The NCHS Research Ethics Review Board approved all protocols and each participant provided informed consent [34]. Data from the occupational questionnaire, interview, examination, and accelerometry components of NHANES 2003–2004 were used in this analysis. Subsequent NHANES cycles did not use similar occupational categories so the analysis herein is necessarily limited to the 2003–2004 cycle.

### Study population

From a potential sample of 2904 participants aged 20–60 years with complete data for all indicated variables (see Table 2 below), participants who reported working at a job or business but not at work (*n* = 114), going to school or retired (*n* = 354), having limitations keeping them from working (*n* = 280), those who did not report an occupational category (*n* = 231), and those who reported less than full-time work (<35 h/week) [35] were excluded (*n* = 351) because of the uncertainty in their employment, or mobility status during the activity monitoring period [36]. Keeping with previous analysis, participants with less than 4 valid days (of ≥10 h/day of wear time) of accelerometer data [37] were also excluded (*n* = 462). The analysis sample ultimately comprised 1112 adults employed full-time in one of 40 occupational categories and with at least 4 days of valid accelerometer data.

**Table 2:** Demographic characteristics by OA groupings in the National Health and Examination Survey 2003–2004 (*n* = 1112)

Characteristic	High OA (*N* = 289)	Intermediate OA (*N* = 300)	Low OA (*N* = 523)	*p*-value*
Age (years), M (SE)	39.3 (0.88)	39.6 (0.74)	42.0 (0.50)	0.009^a,c^
BMI, M (SE)	27.5 (0.57)	28.9 (0.48)	28.2 (0.31)	0.15
Race/ethnicity, %				
Non-Hispanic white	63.6	69.4	77.9	<0.0001^a,c^
Non-Hispanic black	8.3	9.7	8.9	
Mexican American	16.9	11.4	4.1	
Other	11.1	9.6	9.2	
Sex				
Men	86.8	62.1	42.3	<0.001^a,b,c^
Women	13.2	37.9	57.7	
Marital Status				
Married/living with partner	74.5	69.6	69.4	0.2
Single/not living with partner	25.5	30.4	30.6	
Education, %				
<High school	23.5	13.1	2.8	<0.001^a,b,c^
High school	33.3	38.9	16.6	
>High school	43.2	48.0	80.6	
Household Income, %				
<25K	16.5	16.1	5.1	<0.001^a,c^
25–<45K	23.5	31.6	13.1	
45–<75K	33.9	25.7	28.6	
75K+	22	21.5	49.5	
Missing	4.1	5.1	3.7	
Smoking, %				<0.001^a,b,c^
Never	42.9	58.1	58.4	
Former	25.2	12.9	24.4	
Current	31.9	29.0	17.2	
Wear time (min/day), M (SE)	889.5 (7.93)	884.8 (6.99)	884.7 (4.16)	0.89

*High OA* high occupational activity, *Intermediate OA* intermediate occupational activity, *Low OA* low occupational activity, *M* mean, *SE* standard error. *p values for overall group comparisons. Pairwise comparisons: ^a^High OA significantly different from Low OA, ^b^High OA significantly different from Intermediate OA, ^c^Intermediate OA significantly different from Low OA (*p* < 0.05)

### Accelerometry

In 2003 NHANES added the PA monitor component to objectively assess participants greater than 6 years of age. NHANES participants received standardized instructions to wear the uniaxial ActiGraph AM-7164 accelerometer (ActiGraph, Fort Walton Beach, FL) over the right hip attached by an elasticized belt for seven consecutive days, and to remove the monitor during sleeping, bathing, and other aquatic activities. The ActiGraph AM-7164 assesses accelerations ranging from 0.05 to 2.0 g that are band limited with a frequency response of 0.25–2.5 Hz. It was programmed to record information in 1 min epochs and measured vertical acceleration transformed to “activity counts/minute,” a proprietary unit of movement and its intensity. After 7 days participants returned the accelerometers to the NHANES data center by pre-paid mail. Prior to its release, the accelerometer data was examined by NHANES staff for unreasonable values and to confirm that instruments remained calibrated. Unreliable data were clearly marked and not used in this analysis. Additional details about the data collection protocol are available on-line at http://www.cdc.gov/nchs/data/nhanes/meccomp.pdf [38].

Data processing followed previously established standards [37]. Specifically, non-wear time was defined as ≥60 consecutive minutes of zero activity counts/minute, allowing for up to 2 min with activity counts/minute between 0 and 100 [37]. To determine valid days (≥10 h/day of wear time), non-wear time was subtracted from 24 h [39]. For participants with at least 4 days, (week or weekend day, consecutive or not) total activity counts/day (TAC) [40] and wear time mean activity counts/minute (indicators of PA volume that capture varying movement intensities throughout the day) were calculated for each day. Accelerometer wear time data were also classified into activity intensity levels using cut points previously established for NHANES (0–99 counts = sedentary; 100–759 counts = light; 760–2019 counts = lifestyle; ≥ 2020 counts = moderate-to-vigorous PA [MVPA]) [37, 41]. The proportion of time in each activity intensity level was determined by dividing minutes spent in each intensity by minutes of wear time. Steps/day were not released for the 2003–2004 NHANES cycle, so they are not considered in this analysis.

### Population estimates of PA and SB for occupational categories and establishing OA groupings

Mean and standard error (SE) of the six accelerometer-derived variables (TAC, activity counts/minute, proportion of wear time spent in MVPA, lifestyle, light, and SB) accumulated during an average day were calculated for the individuals within each of the 40 occupational categories. The relative standard error for each variable was less than 30 % in accordance with NCHS standards for reporting, unless otherwise noted [42]. Analyses were conducted using SAS software (Research Triangle Park, NC). To account for the complex sampling design utilized by the NHANES, a 2-year sampling weight was calculated and used for analyses following the recommended guidelines from the NCHS.

The 40 occupational categories from NHANES 2003–2004 were ranked (1–40) in ascending order according to each accelerometer-derived variable, except for SB, which was ranked in descending order. A high rank (e.g., 1) was reflective of having greater amounts of OA, while a low rank (e.g., 40) indicated lower amounts of OA. By summing the rank of all six accelerometer-derived variables, a summary score was assigned to each occupational category. Occupational categories were subsequently ranked in ascending order by their summary scores and three mutually exclusive accelerometer-determined OA groupings were created by splitting the ordered summary scores into tertiles: 1) high OA (top tertile of the summary scores, *n* = 13), 2) intermediate OA (middle tertile, *n* = 13), and 3) low OA (bottom tertile, *n* = 14). Considering that the majority of jobs require little OA we chose to allocate 14 occupational categories to the low OA group [20, 21]. Results are presented for men and women by occupational category and OA grouping.

Differences in the characteristics of high OA, intermediate OA, and low OA individuals were analyzed by chi-square test (categorical variables), and analysis of variance (ANOVA) (continuous variables). Adjusted means and standard error (SE) were calculated for accelerometer-derived PA variables and wear time, and ANOVA with Bonferroni correction (alpha = 0.05/7) were used to compare PA variables between men and women in high OA, intermediate OA, and low OA groupings. For the purposes of a comparison other than between OA groupings, TAC of men and women within each OA grouping were compared to age-matched sex-specific population-referenced TAC percentiles (25th, 50th, and 75th) previously determined from NHANES 2003–2006 [40]. Sensitivity analyses were conducted comparing accelerometer-derived PA variables (activity counts/minute, proportion of wear time spent in MVPA, lifestyle, light, and SB) between high, intermediate, and low OA groupings during traditional working hours (9 am–5 pm) and after work (5–10 pm) using ANOVA with Bonferroni correction (alpha = 0.05/5).

## Results

### Characteristics of adults by OA groupings

A large number of adults were excluded from the analytic sample because of inclusion criteria. Compared to the analytical sample (*n* = 1112), those excluded from the study (*n* = 1792) were significantly younger, had lower wear time, accumulated significantly less activity counts/minute and TAC, spent a lower proportion of time in MVPA, lifestyle, and light, and more time sedentary, were more likely to have less than a high school education, to be non-Hispanic black, female, current smokers, single, and lower income (<25 K). We compared subgroups of excluded participants to identify any specific biases—for instance excluded part-time workers were younger, worked less hours, had less lifestyle PA, accelerometer wear time, and TAC than full-time workers. Some occupational categories ‘personal service occupations’, ‘sales workers, retail and personal’, ‘private household occupations’, and ‘waiters and waitresses’ had more part-time than full-time workers. Excluded participants with less than 4 valid days of accelerometer data [37] were younger, had less accelerometer wear time, and total counts. In an effort to maximize generalizability we decided restrict our analysis to full-time workers with good accelerometer wear time compliance.

There were significant differences in sex, age, race/ethnicity, education, income, and smoking status between OA groupings (Table 2). A greater proportion of those grouped as having high and intermediate OA were men. For example, only 13 % of the high OA group were women, whereas 58 % of the low OA group were women. On average, participants grouped as having low OA were significantly older, more educated, had higher household income, and were not current smokers compared to those who were grouped as having high or intermediate OA. A higher proportion of Mexican Americans worked in high and intermediate OA occupations than in low OA occupations. There were no significant differences in BMI, marital status, or accelerometer wear time (884.93 min/day) between any OA groupings.

Tables 4, 5, 6, 7, 8 and 9 highlight the number of men and women sampled from each occupational category. Several occupational categories were represented exclusively by one sex. ‘Related agricultural, forestry, and fishing (*n* = 21)’, ‘construction laborers (*n* = 9)’, ‘other mechanics and repairers (*n* = 37)’, and ‘vehicle and mobile equipment mechanics and repairers (*n* = 15)’ were occupational categories with 100 % men; while ‘private household (*n* = 9)’, ‘records processing (*n* = 33)’, and ‘secretaries, stenographers, and typists (*n* = 21)’ were only represented by women. Sixteen of the 40 occupational categories were represented by less than 20 individuals, and seven occupational categories were represented by less than 10 individuals. In the smallest occupational category, ‘laborers, except construction’ there were only data available for three individuals (1 man, 2 women). Because of the small numbers and sex imbalances of certain occupational categories we chose not to run statistical comparisons within or between the 40 occupational categories.

### Low, intermediate, and high OA

Mean and standard error (SE) for the six accelerometer-derived variables in rank order for each of the 40 occupational categories are presented in Tables 4, 5, 6, 7, 8 and 9. ‘Secretaries, stenographers, and typists’ had the lowest TAC, and activity counts/minute followed by ‘records processing occupations.’ ‘Related agricultural, forestry, and fishing occupations’ had the highest TAC, activity counts/minute, and the largest proportion of MVPA (8 %). ‘Engineers, architects and scientists’ had the largest proportion of monitored time spent in SB (65 %), and the smallest proportion of time spent in light (23 %) and lifestyle PA (8 %). Conversely, ‘waiters and waitresses’ had the smallest proportion of time spent in SB (40 %), and largest proportion of time spent in light PA (43 %).

Table 3 presents an overall summary of the six accelerometer-derived variables assembled in Tables 4, 5, 6, 7, 8 and 9. Specifically, the 40 occupational categories are ranked in ascending order according to their summary score, and the three accelerometer-determined OA groupings derived from the tertile split of the summary score are labelled. The high, and intermediate OA groupings included 13 occupational categories, while 14 occupational categories were assigned to the low OA grouping. The corresponding rank for each accelerometer-derived variable is presented for each occupational category. ‘Secretaries, stenographers, and typists’ and ‘records processing occupations’ were consistently in the lower ranks for most accelerometer-derived variables, while ‘farm and nursery workers’, ‘other helpers, equipment cleaners, hand packagers, and laborers’, ‘construction laborers’, and ‘related agricultural, forestry, and fishing occupations’ were consistently near the top for most accelerometer-derived variables and were the four highest ranked occupational categories according to the summary score.

**Table 3:** Occupational categories ranked by summary score of the accelerometer-derived variables and the corresponding OA groupings

Rank	OA grouping	Occupational category	TAC	Activity counts/min	MVPA %	Lifestyle %	Light %	Sedentary %	Summary score
1	High OA	Farm and nursery workers	5	3	3	3	**14 ↓**	3	**31**
2		Other helpers, cleaners, hand packagers, laborers	8	6	8	6	6	2	**36**
3		Construction laborers	2	2	2	5	**26 ↓**	4	**41**
4		Related agricultural, forestry, and fishing	1	1	1	4	**30 ↓↓**	5	**42**
5		Cleaning and building service occupations	3	4	7	1	**23 ↓**	6	**44**
6		Construction trades	7	5	5	7	**17 ↓**	7	**48**
7		Freight, stock, and material movers (hand)	6	8	9	2	**16 ↓**	9	**50**
8		Farm operators, managers, and supervisors	4	7	4	8	**19 ↓**	10	**52**
9		Textile, apparel, furnishings machine operators	9	9	6	13	**22 ↓**	13	**72**
10		Machine operators, assorted materials	13	11	**22 ↓**	**15 ↓**	4	8	**73**
11		Waiters and waitresses	**16 ↓**	13	**32 ↓↓**	11	1	1	**74**
12		Other mechanics and repairers	12	10	**14 ↓**	12	**18 ↓**	**15 ↓**	**81**
13		Motor vehicle operators	11	**14 ↓**	12	**23 ↓**	10	**17 ↓**	**87**
14	Intermediate OA	Supervisors and proprietors, sales occupations	14	15	24	**10 ↑**	**12 ↑**	14	**89**
15		Fabricators, assemblers, inspectors, and samplers	17	18	15	22	**8 ↑**	18	**98**
16		Other transportation and material moving	**10 ↑**	**12 ↑**	**11 ↑**	18	**32 ↓**	26	**109**
17		Private household occupations	20	16	**10 ↑**	19	24	21	**110**
18		Vehicle and mobile equip. mechanics, repairers	19	19	**29 ↓**	16	**11 ↑**	16	**110**
19		Material recording, scheduling, distributing clerks	15	17	21	14	**28 ↓**	23	**118**
20		Cooks	25	24	**33 ↓**	25	**3 ↑**	**12 ↑**	**122**
21		Miscellaneous food preparation and service	**32 ↓**	22	**35 ↓**	24	**2 ↑**	**11 ↑**	**126**
22		Extractive and precision production occupations	18	20	25	21	21	24	**129**
23		Laborers, except construction	22	26	**40 ↓**	**9 ↑**	**13 ↑**	20	**130**
24		Sales workers, retail and personal services	21	21	26	20	20	22	**130**
25		Health service occupations	**31 ↓**	25	**34 ↓**	17	**7 ↑**	19	**133**
26		Sales reps., finance, business, & commodities	23	23	18	26	**35 ↓**	**31 ↓**	**156**
27	Low OA	Technicians and related support occupations	**26 ↑**	27	**16 ↑**	37	33	32	**171**
28		Information clerks	35	37	36	36	**5 ↑↑**	**25 ↑**	**174**
29		Health diagnosing, assessing and treating	33	34	31	34	**15 ↑**	28	**175**
30		Executive, administrators, and managers	29	29	**23 ↑**	28	34	33	**176**
31		Writers, artists, entertainers, and athletes	27	30	**17 ↑**	31	36	36	**177**
32		Personal service occupations	38	38	39	30	**9 ↑↑**	27	**181**
33		Management related occupations	**24 ↑**	28	**19 ↑**	33	39	39	**182**
34		Teachers	30	32	27	35	29	30	**183**
35		Protective service occupations	34	33	**20 ↑**	27	38	37	**189**
36		Engineers, architects and scientists	28	31	**13 ↑↑**	40	40	40	**192**
37		Miscellaneous administrative support occupations	37	35	28	32	31	29	**192**
38		Other professional specialty occupations	36	36	30	29	37	38	**206**
39		Records processing occupations	39	39	37	38	27	35	**215**
40		Secretaries, stenographers, and typists	40	40	38	39	**25 ↑**	34	**216**

Rank, the 40 occupational categories were ranked in ascending order according to the summary score, a higher rank (e.g., 1) is reflective of having greater amounts of OA (occupational activity); OA Grouping, tertile splits of the summary scores were used to establish three accelerometer determined OA groupings: high OA, intermediate OA, and low OA; Occupational category, based on the 1980 U.S. Census Bureau Classification Coding System; TAC, rank according to total activity counts; Activity counts/min, rank according to activity counts per minute; MVPA%, rank according to the proportion of time spent in moderate-to-vigorous physical activity (≥2020 counts); Lifestyle %, rank according to the proportion of time spent in lifestyle intensity physical activity (760–2019 counts); Light %, rank according to the proportion of time spent in light intensity physical activity (100–759 counts); Sedentary %, rank according to the proportion of time spent in sedentary intensity activity (0–99 counts). Sedentary % reverse coded so that 1 = least sedentary; Summary score, sum of the rank of all six accelerometer-derived variables for each occupational category. Bolded values = higher (↑) or lower (↓) values for an accelerometer-derived variable relative to their OA grouping; (↑ or ↓) = jumped one OA grouping relative to their OA grouping, (↑↑ or ↓↓) = jumped two OA groupings relative to their OA grouping

**Table 4:** Ranking for daily TAC by occupational category and by sex: NHANES 2003–2004 (*n* = 1112)

Rank	Occupational category	Overall *n*	TAC M (SE)	Men *n*	TAC M (SE)	Women *n*	TAC M (SE)
1	Related agricultural, forestry, and fishing	21	485,767 (29,836)	21	485,767 (29,836)	0	-
2	Construction laborers	9	471,863 (54,189)	9	471,863 (54,189)	0	-
3	Cleaning and building service occupations	23	450,084 (30,591)	15	483,758 (51,323)	8	397,142 (79,167)
4	Farm operators, managers, and supervisors	5	436,923 (295.8)	4	448,050 (4261)	1	329,570 (0)
5	Farm and nursery workers	6	434,533 (72,549)	5	479,218 (61,203)	1	168,400 (0)
6	Freight, stock, and material movers (hand)	14	425,193 (22,221)	11	425,427 (28,972)	3	424,461 (5927)
7	Construction trades	58	424,700 (19,785)	57	426,154 (20,224)	1	256,600 (0)
8	Other helpers, cleaners, packagers, laborers	18	422,397 (43,719)	12	494,944 (51,212)	6	301,419 (8065)
9	Textile, apparel, furnishings machine operators	8	389,395 (72,764)	3	430,829 (54,246)	5	378,150 (90,086)
10	Other transportation and material moving	21	363,428 (19,964)	20	369,009 (20,866)	1	279,710 (0)
11	Motor vehicle operators	61	358,903 (21,335)	59	365,130 (21,238)	2	219,442 (61,238)
12	Other mechanics and repairers	37	358,301 (15,423)	37	358,301 (15,423)	0	-
13	Machine operators, assorted materials	22	356,234 (37,952)	17	386,340 (35,424)	5	214,122 (22,970)
14	Supervisors and proprietors, sales occupations	27	346,590 (35,260)	19	371,350 (43,575)	8	291,278 (35,530)
15	Material recording, scheduling, distributing clerks	19	334,085 (60,295)	12	358,570 (85,201)	7	272,997 (41,793)
16	Waiters and waitresses	7	332,931 (32,540)	2	503,971 (73,138)	5	304,425 (41,551)
17	Fabricators, assemblers, inspectors, and samplers	28	332,848 (17,945)	16	378,576 (28,778)	12	248,454 (15,706)
18	Extractive and precision production occupations	47	327,915 (17,333)	38	336,678 (20,915)	9	295,118 (60,034)
19	Vehicle and mobile equip. mechanics, repairers	15	320,773 (40,082)	15	320,773 (40,082)	0	-
20	Private household occupations	9	320,474 (84,755)	0	-	9	320,474 (84,755)
21	Sales workers, retail and personal services	26	314,124 (18,394)	12	339,663 (23,099)	14	291,893 (21,422)
22	Laborers, except construction	3	305,925 (71,578)	1	174,290 (0)	2	409,405 (2793)
23	Sales reps., finance, business, & commodities	29	303,648 (23,216)	16	343,308 (42,571)	13	237,490 (17,916)
24	Management related occupations	49	299,487 (16,539)	23	335,330 (28,517)	26	258,586 (14,012)
25	Cooks	24	298,019 (19,802)	13	314,980 (31,615)	11	262,556 (18,198)
26	Technicians and related support occupations	33	296,734 (17,082)	18	279,416 (22,874)	15	315,969 (30,724)
27	Writers, artists, entertainers, and athletes	15	296,033 (30,519)	9	342,968 (41,433)	6	229,124 (22,602)
28	Engineers, architects and scientists	29	294,668 (33,219)	22	300,829 (38,693)	7	267,305 (28,429)
29	Executive, administrators, and managers	112	293,657 (10,403)	67	325,623 (12,281)	45	239,623 (15,811)
30	Teachers	40	292,993 (23,268)	11	373,185 (44,851)	29	267,744 (23,133)
31	Health service occupations	38	290,882 (22,132)	7	330,900 (35,382)	31	280,704 (19,070)
32	Miscellaneous food preparation and service	14	288,423 (20,262)	6	294,952 (22,086)	8	285,417 (30,348)
33	Health diagnosing, assessing and treating	42	284,889 (13,572)	8	290,655 (22,823)	34	283,369 (16,371)
34	Protective service occupations	13	281,516 (33,814)	10	278,523 (39,317)	3	298,215 (22,129)
35	Information clerks	18	280,789 (44,557)	4	215,013 (19,044)	14	291,417 (50,557)
36	Other professional specialty occupations	39	279,639 (16,847)	16	296,210 (31,331)	23	262,859 (17,062)
37	Miscellaneous administrative support occupations	65	277,748 (14,067)	16	361,957 (46,240)	49	253,329 (16,315)
38	Personal service occupations	14	246,259 (19,502)	3	316,398 (28,738)	11	215,592 (12,896)
39	Records processing occupations	33	243,927 (11,345)	0	-	33	243,927 (11,345)
40	Secretaries, stenographers, and typists	21	223,662 (15,790)	0	-	21	223,662 (15,790)

*TAC* total activity counts, *M* mean, *SE* standard error

**Table 5:** Ranking for activity counts/minute by occupational category and by sex: NHANES 2003–2004 (*n* = 1112)

Rank	Occupational category	Overall *n*	Activity counts/min M (SE)	Men *n*	Activity counts/min M (SE)	Women *n*	Activity counts/min M (SE)
1	Related agricultural, forestry, and fishing	21	567.4 (34.4)	21	567.4 (34.4)	0	-
2	Construction laborers	9	551.4 (47)	9	551.4 (47)	0	-
3	Farm and nursery workers	6	509.3 (76.3)	5	551.6 (68.1)	1	257.4 (0)
4	Cleaning and building service occupations	23	496.4 (31)	15	530.9 (49.6)	8	442 (83.8)
5	Construction trades	58	487.9 (19.9)	57	489.6 (20.4)	1	283 (0)
6	Other helpers, cleaners, packagers, laborers	18	483.4 (40.3)	12	554 (52.4)	6	365.8 (17)
7	Farm operators, managers, and supervisors	5	479.8 (12.1)	4	491.6 (17.5)	1	366.8 (0)
8	Freight, stock, and material movers (hand)	14	465.8 (40.2)	11	457 (51.2)	3	493.4 (25.2)
9	Textile, apparel, furnishings machine operators	8	454.4 (90.2)	3	518.6 (67.4)	5	437 (113.5)
10	Other mechanics and repairers	37	410.6 (19.7)	37	410.6 (19.7)	0	-
11	Machine operators, assorted materials	22	405.4 (34.6)	17	437.7 (29.6)	5	253 (19.2)
12	Other transportation and material moving	21	399.9 (20.3)	20	407.4 (20.4)	1	287.4 (0)
13	Waiters and waitresses	7	398.8 (36.3)	2	598.4 (78.5)	5	365.6 (42.1)
14	Motor vehicle operators	61	398.2 (28.1)	59	407.3 (28.7)	2	193.3 (26.9)
15	Supervisors and proprietors, sales occupations	27	393.4 (35.8)	19	416.9 (46)	8	340.8 (40.1)
16	Private household occupations	9	391 (98.1)	0	-	9	391 (98.1)
17	Material recording, scheduling, distributing clerks	19	386.8 (75.8)	12	426.5 (105.6)	7	287.8 (35.3)
18	Fabricators, assemblers, inspectors, and samplers	28	380 (34.4)	16	435.2 (46.7)	12	278.1 (18.2)
19	Vehicle and mobile equip. mechanics, repairers	15	374.5 (42)	15	374.5 (42)	0	-
20	Extractive and precision production occupations	47	362.4 (16.4)	38	367.3 (15.1)	9	344 (69.2)
21	Sales workers, retail and personal services	26	360.2 (19.8)	12	383 (21.8)	14	340.4 (28.7)
22	Miscellaneous food preparation and service	14	348.2 (27.6)	6	351.6 (39.2)	8	346.7 (34.5)
23	Sales reps., finance, business, & commodities	29	348 (24.5)	16	387.8 (45.1)	13	281.6 (23.1)
24	Cooks	24	341.9 (22.8)	13	354.3 (31)	11	316.1 (33.6)
25	Health service occupations	38	339.6 (25.8)	7	385.8 (41.2)	31	327.9 (21.8)
26	Laborers, except construction	3	339.2 (65.1)	1	219.4 (0)	2	433.4 (32)
27	Technicians and related support occupations	33	338.9 (19.2)	18	315.3 (26.2)	15	365 (35)
28	Management related occupations	49	337.6 (16.1)	23	374.5 (25.8)	26	295.6 (16.7)
29	Executive, administrators, and managers	112	335.5 (11.7)	67	370.4 (13.5)	45	276.4 (18.3)
30	Writers, artists, entertainers, and athletes	15	335.3 (33.1)	9	385 (44.7)	6	264.3 (26.7)
31	Engineers, architects and scientists	29	332.3 (35.8)	22	336.6 (42.1)	7	313.5 (34.9)
32	Teachers	40	328.8 (29.8)	11	418.5 (61.7)	29	300.6 (28.8)
33	Protective service occupations	13	327 (40.2)	10	326.7 (47)	3	328.6 (5.7)
34	Health diagnosing, assessing and treating	42	326.1 (14.9)	8	348.2 (27.2)	34	320.3 (19.4)
35	Miscellaneous administrative support occupations	65	320.7 (17)	16	400.1 (56.1)	49	297.6 (19.6)
36	Other professional specialty occupations	39	319.3 (20.1)	16	336.2 (37.8)	23	302.1 (15.6)
37	Information clerks	18	312.6 (44.4)	4	262.9 (4.4)	14	320.6 (51.1)
38	Personal service occupations	14	287.5 (24.7)	3	365.3 (30.7)	11	253.5 (21.8)
39	Records processing occupations	33	269.6 (11.6)	0	-	33	269.6 (11.6)
40	Secretaries, stenographers, and typists	21	268.7 (19.2)	0	-	21	268.7 (19.2)

*M* mean, *SE* standard error

**Table 6:** Ranking for proportion of time in MVPA by occupational category and by sex: NHANES 2003–2004 (*n* = 1112)

Rank	Occupational category	Overall *n*	MVPA M (SE)	Men *n*	MVPA M (SE)	Women *n*	MVPA M (SE)
1	Related agricultural, forestry, and fishing	21	7.7 (1)	21	7.7 (1)	0	-
2	Construction laborers	9	7.4 (1)	9	7.4 (1)	0	-
3	Farm and nursery workers	6	5.7 (1.3)	5	6.4 (1.2)	1	1.5 (0)
4	Farm operators, managers, and supervisors	5	5.6 (0.8)	4	5.9 (0.9)	1	3.2 (0)
5	Construction trades	58	5.6 (0.4)	57	5.6 (0.4)	1	1.2 (0)
6	Textile, apparel, furnishings machine operators	8	5.4 (1.5)	3	6.1 (0.2)	5	5.2 (1.9)
7	Cleaning and building service occupations	23	5.1 (0.4)	15	6 (0.8)	8	3.7 (1)
8	Other helpers, cleaners, packagers, laborers	18	4.8 (0.8)	12	5.8 (1.2)	6	3 (0.2)
9	Freight, stock, and material movers (hand)	14	4.4 (0.6)	11	4.5 (0.8)	3	4 (0.8)
10	Private household occupations^a^	9	4.3 (1.5)	0	-	9	4.3 (1.5)
11	Other transportation and material moving	21	4.2 (0.3)	20	4.3 (0.3)	1	2.5 (0)
12	Motor vehicle operators	61	4.1 (0.5)	59	4.2 (0.5)	2	0.7 (0.3)
13	Engineers, architects and scientists	29	4 (0.7)	22	4.1 (0.8)	7	3.3 (0.6)
14	Other mechanics and repairers	37	4 (0.5)	37	4 (0.5)	0	-
15	Fabricators, assemblers, inspectors, and samplers	28	3.6 (0.5)	16	4.5 (0.6)	12	1.9 (0.4)
16	Technicians and related support occupations	33	3.6 (0.4)	18	3.6 (0.6)	15	3.7 (0.7)
17	Writers, artists, entertainers, and athletes	15	3.5 (0.7)	9	4.7 (1)	6	1.9 (0.3)
18	Sales reps., finance, business, & commodities	29	3.5 (0.6)	16	4.6 (1)	13	1.8 (0.5)
19	Management related occupations	49	3.5 (0.4)	23	4.2 (0.5)	26	2.8 (0.4)
20	Protective service occupations	13	3.4 (1)	10	3.7 (1.1)	3	2 (0.4)
21	Material recording, scheduling, distributing clerks^a^	19	3.3 (1)	12	3.7 (1.5)	7	2.2 (0.4)
22	Machine operators, assorted materials	22	3.3 (0.6)	17	3.7 (0.6)	5	1.6 (0.4)
23	Executive, administrators, and managers	112	3.2 (0.2)	67	3.9 (0.3)	45	2 (0.3)
24	Supervisors and proprietors, sales occupations	27	3.1 (0.5)	19	3.7 (0.7)	8	1.9 (0.4)
25	Extractive and precision production occupations	47	3.1 (0.3)	38	3.3 (0.3)	9	2.1 (0.6)
26	Sales workers, retail and personal services	26	3.1 (0.3)	12	3.8 (0.4)	14	2.4 (0.4)
27	Teachers	40	3 (0.5)	11	4.2 (0.9)	29	2.6 (0.5)
28	Miscellaneous administrative support occupations	65	3 (0.3)	16	4.9 (1.3)	49	2.4 (0.4)
29	Vehicle and mobile equip. mechanics, repairers	15	2.9 (0.7)	15	2.9 (0.7)	0	-
30	Other professional specialty occupations	39	2.9 (0.3)	16	3.4 (0.5)	23	2.5 (0.2)
31	Health diagnosing, assessing and treating	42	2.7 (0.3)	8	3.2 (0.5)	34	2.6 (0.3)
32	Waiters and waitresses^a^	7	2.5 (0.9)	2	5.8 (0.8)	5	1.9 (0.8)
33	Cooks	24	2.2 (0.4)	13	2.5 (0.4)	11	1.5 (0.5)
34	Health service occupations	38	2.1 (0.4)	7	3.4 (0.8)	31	1.8 (0.3)
35	Miscellaneous food preparation and service	14	2.1 (0.4)	6	2.3 (1)	8	2 (0.4)
36	Information clerks^a^	18	1.9 (0.6)	4	2.5 (0.5)	14	1.8 (0.7)
37	Records processing occupations	33	1.8 (0.2)	0	-	33	1.8 (0.2)
38	Secretaries, stenographers, and typists	21	1.7 (0.4)	0	-	21	1.7 (0.4)
39	Personal service occupations	14	1.6 (0.4)	3	3.1 (0.5)	11	1 (0.1)
40	Laborers, except construction	3	1.3 (0.3)	1	0.9 (0)	2	1.6 (0.3)

*MVPA* moderate-to-vigorous physical activity (≥2020 counts), *M* mean, *SE* standard error. ^a^The relative standard deviation is greater than the 30 % standard for reporting required by NCHS indicating the value may be unreliable due to large variance

**Table 7:** Ranking for proportion of time in lifestyle activity by occupational category and by sex: NHANES 2003–2004 (*n* = 1112)

Rank	Occupational category	Overall *n*	Lifestyle activity M (SE)	Men *n*	Lifestyle activity M (SE)	Women *n*	Lifestyle activity M (SE)
1	Cleaning and building service occupations	23	18.1 (1.9)	15	18.8 (2.2)	8	16.9 (4.7)
2	Freight, stock, and material movers (hand)	14	17.2 (2)	11	16.6 (2.5)	3	19 (1)
3	Farm and nursery workers	6	17.1 (2.6)	5	18.4 (2.2)	1	9.1 (0)
4	Related agricultural, forestry, and fishing	21	17 (0.8)	21	17 (0.8)	13	9.9 (0.8)
5	Construction laborers	9	16.8 (1.4)	9	16.8 (1.4)	0	-
6	Other helpers, cleaners, hand packagers, laborers	18	16.7 (1.6)	12	19.8 (1.9)	6	11.5 (1.1)
7	Construction trades	58	16.4 (0.6)	57	16.4 (0.6)	1	11.1 (0)
8	Farm operators, managers, and supervisors	5	15.9 (0.7)	4	16.4 (0.7)	1	11.8 (0)
9	Laborers, except construction	3	15.2 (3.8)	1	8.3 (0)	2	20.7 (2.3)
10	Supervisors and proprietors, sales occupations	27	14.8 (1.5)	19	15.7 (1.8)	8	12.8 (2.1)
11	Waiters and waitresses	7	14.4 (2.1)	2	23.4 (3.6)	5	12.9 (2.6)
12	Other mechanics and repairers	37	14.3 (0.6)	37	14.3 (0.6)	0	-
13	Textile, apparel, furnishings machine operators	8	14.3 (3.2)	3	17.6 (6)	5	13.4 (3.6)
14	Material recording, scheduling, distributing clerks	19	14.2 (3.5)	12	16.1 (4.7)	7	9.6 (1.3)
15	Machine operators, assorted materials	22	14.2 (1.4)	17	15.8 (1.1)	5	6.9 (1)
16	Vehicle and mobile equip. mechanics, repairers	15	13.9 (1.6)	15	13.9 (1.6)	0	-
17	Health service occupations	38	13 (1)	7	14.1 (1.4)	31	12.7 (1)
18	Other transportation and material moving	21	12.9 (0.8)	20	13.1 (0.8)	1	9.8 (0)
19	Private household occupations	9	12.9 (3.5)	0	-	9	12.9 (3.5)
20	Sales workers, retail and personal services	26	12.8 (1.1)	12	12.9 (1.1)	0	-
21	Extractive and precision production occupations	47	12.8 (0.9)	38	12.6 (0.7)	9	13.6 (4)
22	Fabricators, assemblers, inspectors, and samplers	28	12.6 (1.6)	16	14.7 (2.1)	12	8.6 (0.7)
23	Motor vehicle operators	61	12.4 (0.9)	59	12.7 (0.9)	2	6.3 (1.1)
24	Miscellaneous food preparation and service	14	12 (1.5)	6	12.2 (1.4)	8	11.9 (2)
25	Cooks	24	10.9 (1.1)	13	11.2 (1.3)	11	10.2 (1.9)
26	Sales reps., finance, business, & commodities	29	10.6 (0.9)	16	11 (1.6)	14	12.8 (1.6)
27	Protective service occupations	13	10.4 (0.8)	10	10.1 (0.9)	3	12.4 (0.8)
28	Executive, administrators, and managers	112	10.3 (0.4)	67	11.2 (0.6)	45	8.8 (0.5)
29	Other professional specialty occupations	39	10.2 (0.8)	16	10.6 (1.4)	23	9.9 (0.8)
30	Personal service occupations	14	10.2 (1.1)	3	13.6 (1.5)	11	8.7 (1)
31	Writers, artists, entertainers, and athletes	15	10 (1)	9	11 (1.3)	6	8.6 (1)
32	Miscellaneous administrative support occupations	65	9.8 (0.5)	16	12.3 (1.4)	49	9.1 (0.6)
33	Management related occupations	49	9.8 (0.5)	23	10.8 (1)	26	8.6 (0.7)
34	Health diagnosing, assessing and treating	42	9.6 (0.5)	8	10.2 (1.4)	34	9.5 (0.5)
35	Teachers	40	9.6 (0.7)	11	12.2 (0.7)	29	8.8 (1)
36	Information clerks	18	9.4 (1)	4	7.7 (0.9)	14	9.7 (1.1)
37	Technicians and related support occupations	33	9.3 (1)	18	8.9 (1.3)	15	9.8 (1.1)
38	Records processing occupations	33	9.1 (0.4)	0	-	33	9.1 (0.4)
39	Secretaries, stenographers, and typists	21	9 (0.6)	0	-	21	9 (0.6)
40	Engineers, architects and scientists	29	8 (0.6)	22	8 (0.7)	7	8.2 (0.6)

Lifestyle activity, 760–2019 counts; *M* mean, *SE* standard error

**Table 8:** Ranking for proportion of time in light activity by occupational category and by sex: NHANES 2003–2004 (*n* = 1112)

Rank	Occupational category	Overall *n*	Light activity M (SE)	Men *n*	Light activity M (SE)	Women *n*	Light activity M (SE)
1	Waiters and waitresses	7	43.4 (3.4)	2	32.3 (1.6)	5	45.2 (2.6)
2	Miscellaneous food preparation and service	14	39.2 (2.3)	6	38.3 (3.2)	8	39.6 (2.8)
3	Cooks	24	39 (2.1)	13	36.9 (2.3)	11	43.3 (1)
4	Machine operators, assorted materials	22	36.6 (1.5)	17	36.4 (1.6)	5	37.4 (4.8)
5	Information clerks	18	36.1 (1.3)	4	26.8 (1.7)	14	37.6 (0.9)
6	Other helpers, cleaners, hand packagers, laborers	18	36.1 (1.8)	12	33.6 (1.9)	6	40.2 (2.1)
7	Health service occupations	38	34.6 (0.7)	7	31.5 (0.7)	31	35.3 (0.9)
8	Fabricators, assemblers, inspectors, and samplers	28	33.7 (2)	16	33.6 (2.6)	12	34 (3.1)
9	Personal service occupations	14	33.7 (2)	3	30.4 (0.5)	11	35.1 (2.7)
10	Motor vehicle operators	61	33.6 (1.1)	59	33.8 (1.2)	2	28.7 (1.6)
11	Vehicle and mobile equip. mechanics, repairers	15	33.3 (1.4)	15	33.3 (1.4)	0	-
12	Supervisors and proprietors, sales occupations	27	33.1 (1.6)	19	31.1 (1.5)	8	37.6 (2.9)
13	Laborers, except construction	3	33 (3.9)	1	26.1 (0)	2	38.4 (3.3)
14	Farm and nursery workers	6	32.5 (2.1)	5	32.2 (2.6)	1	34.7 (0)
15	Health diagnosing, assessing and treating	42	32.5 (1)	8	34.5 (3.5)	34	31.9 (1)
16	Freight, stock, and material movers (hand)	14	32.3 (1.4)	11	31.2 (1.9)	3	35.9 (0.9)
17	Construction trades	58	32.3 (0.7)	57	32.3 (0.7)	1	32.6 (0)
18	Other mechanics and repairers	37	32.2 (1)	37	32.2 (1)	0	-
19	Farm operators, managers, and supervisors	5	32.1 (1)	4	31.6 (1.3)	1	36.7 (0)
20	Sales workers, retail and personal services	26	32.1 (0.9)	12	31.1 (1)	14	32.9 (1.7)
21	Extractive and precision production occupations	47	31.7 (0.8)	38	31.2 (0.7)	9	33.8 (1.8)
22	Textile, apparel, furnishings machine operators	8	31.4 (2)	3	29.2 (3.7)	5	32 (2.1)
23	Cleaning and building service occupations	23	31.3 (0.6)	15	32 (0.8)	8	30.1 (1.3)
24	Private household occupations	9	31.2 (4.9)	0	-	9	31.2 (4.9)
25	Secretaries, stenographers, and typists	21	30.5 (1.9)	0	-	21	30.5 (1.9)
26	Construction laborers	9	30.5 (1.6)	9	30.5 (1.6)	0	-
27	Records processing occupations	33	30.2 (1.3)	0	-	33	30.2 (1.3)
28	Material recording, scheduling, distributing clerks	19	30.2 (0.9)	12	29.4 (1.1)	7	32.2 (1.8)
29	Teachers	40	29.9 (1.2)	11	29.2 (1.5)	29	30.1 (1.6)
30	Related agricultural, forestry, and fishing	21	29.9 (1.7)	21	29.9 (1.7)	0	-
31	Miscellaneous administrative support occupations	65	29.7 (0.9)	16	25.3 (1.2)	49	30.9 (1.1)
32	Other transportation and material moving	21	28.8 (1.7)	20	28.9 (1.8)	1	27.4 (0)
33	Technicians and related support occupations	33	28.6 (1.8)	18	24.7 (2.1)	15	33 (1.8)
34	Executive, administrators, and managers	112	27.8 (0.8)	67	27.2 (1.2)	45	28.9 (0.8)
35	Sales reps., finance, business, & commodities	29	27.7 (1.3)	16	25.8 (1.4)	13	30.7 (1.2)
36	Writers, artists, entertainers, and athletes	15	27.5 (1.4)	9	26.7 (1.7)	6	28.6 (2.1)
37	Other professional specialty occupations	39	26.9 (1.1)	16	25.7 (1.3)	23	28.1 (1.1)
38	Protective service occupations	13	26.5 (1.8)	10	25.3 (1.7)	3	33 (3.6)
39	Management related occupations	49	26.4 (0.8)	23	25.2 (1.2)	26	27.8 (0.5)
40	Engineers, architects and scientists	29	23 (0.8)	22	22.5 (0.7)	7	25.5 (2.3)

Light activity, 100–759 counts; *M* mean, *SE* standard error

**Table 9:** Ranking for proportion of time in sedentary activity by occupational category and by sex: NHANES 2003–2004 (*n* = 1112)

Rank	Occupational category	Overall *n*	Sedentary activity M (SE)	Men *n*	Sedentary activity M (SE)	Women *n*	Sedentary activity M (SE)
1	Waiters and waitresses	7	39.8 (3.2)	2	38.5 (6)	5	40 (4.3)
2	Other helpers, cleaners, hand packagers, laborers	18	42.4 (1.2)	12	40.7 (1.6)	6	45.3 (1.6)
3	Farm and nursery workers	6	44.7 (5.4)	5	43 (5.5)	1	54.7 (0)
4	Construction laborers	9	45.3 (2)	9	45.3 (2)	0	-
5	Related agricultural, forestry, and fishing	21	45.4 (1.3)	21	45.4 (1.3)	0	-
6	Cleaning and building service occupations	23	45.6 (2.2)	15	43.2 (3.5)	8	49.3 (5.4)
7	Construction trades	58	45.7 (1.3)	57	45.6 (1.3)	1	55.1 (0)
8	Machine operators, assorted materials	22	45.9 (1.5)	17	44.2 (1.5)	5	54.1 (4.7)
9	Freight, stock, and material movers (hand)	14	46.1 (2.9)	11	47.8 (3.8)	3	41 (0.4)
10	Farm operators, managers, and supervisors	5	46.3 (1)	4	46.1 (1)	1	48.3 (0)
11	Miscellaneous food preparation and service	14	46.7 (2.3)	6	47.2 (3.5)	8	46.5 (2.4)
12	Cooks	24	47.9 (2.6)	13	49.3 (3.2)	11	45 (2.1)
13	Textile, apparel, furnishings machine operators	8	48.9 (5.5)	3	47.1 (6.6)	5	49.4 (6.9)
14	Supervisors and proprietors, sales occupations	27	49 (3)	19	49.6 (3.6)	8	47.7 (5)
15	Other mechanics and repairers	37	49.5 (1.2)	37	49.5 (1.2)	0	-
16	Vehicle and mobile equip. mechanics, repairers	15	49.8 (2.9)	15	49.8 (2.9)	0	-
17	Motor vehicle operators	61	50 (1.5)	59	49.3 (1.5)	2	64.3 (3)
18	Fabricators, assemblers, inspectors, and samplers	28	50.1 (3.6)	16	47.2 (5.1)	12	55.5 (3.1)
19	Health service occupations	38	50.4 (1.6)	7	51 (1.9)	31	50.2 (1.8)
20	Laborers, except construction	3	50.5 (7.9)	1	64.7 (0)	2	39.3 (5.3)
21	Private household occupations	9	51.7 (9.1)	0	-	9	51.7 (9.1)
22	Sales workers, retail and personal services	26	52 (1.5)	12	52.1 (1.7)	14	51.9 (2.4)
23	Material recording, scheduling, distributing clerks	19	52.3 (4.4)	12	50.8 (6)	7	56.1 (3.4)
24	Extractive and precision production occupations	47	52.4 (1.2)	38	52.9 (0.9)	9	50.4 (5.3)
25	Information clerks	18	52.5 (1.8)	4	63 (2)	14	50.8 (1.5)
26	Other transportation and material moving	21	54.1 (2.2)	20	53.7 (2.3)	1	60.2 (0)
27	Personal service occupations	14	54.5 (2.5)	3	53 (1.5)	11	55.2 (3.5)
28	Health diagnosing, assessing and treating	42	55.2 (1.1)	8	52.1 (4.4)	34	56.1 (1.1)
29	Miscellaneous administrative support occupations	65	57.5 (1.4)	16	57.5 (3.1)	49	57.5 (1.7)
30	Teachers	40	57.5 (1.7)	11	54.4 (1.7)	29	58.5 (2.4)
31	Sales reps., finance, business, & commodities	29	58.2 (1.9)	16	58.6 (3.1)	13	57.6 (2)
32	Technicians and related support occupations	33	58.5 (2.5)	18	62.9 (3.2)	15	53.6 (2.4)
33	Executive, administrators, and managers	112	58.6 (1.1)	67	57.7 (1.4)	45	60.2 (1.4)
34	Secretaries, stenographers, and typists	21	58.7 (2.3)	0	-	21	58.7 (2.3)
35	Records processing occupations	33	58.8 (1.6)	0	-	33	58.8 (1.6)
36	Writers, artists, entertainers, and athletes	15	58.9 (2.6)	9	57.6 (3.3)	6	60.9 (3)
37	Protective service occupations	13	59.7 (2.4)	10	60.9 (2.6)	3	52.6 (3.8)
38	Other professional specialty occupations	39	59.9 (1.8)	16	60.3 (2.9)	23	59.6 (1.7)
39	Management related occupations	49	60.3 (1.2)	23	59.7 (2.1)	26	60.9 (0.9)
40	Engineers, architects and scientists	29	65 (1.4)	22	65.4 (1.6)	7	63 (3)

Sedentary activity, 0–99 counts; *M* mean, *SE* standard error

Among the three OA groupings most of the individual accelerometer-derived variables aligned closely with the summary scores (Table 3). Scrutinizing the low OA grouping revealed several notable exceptions. For example, some low OA occupational categories (e.g., ‘engineers, architects, and scientists’, ‘technicians and related support occupations’, ‘management related occupations’, ‘executives, administrators, and managers’, ‘protective services’, and ‘writers, artists, entertainers, and athletes’) displayed relatively higher rankings for the proportion of time in MVPA. To be clear, their time in MVPA was higher than what was expected given their relatively lower summary score.

Within each OA grouping men accumulated significantly more TAC, activity counts/minute, and had a greater proportion of time in MVPA than women (Table 10). Within the intermediate OA and low OA groupings, women spent a significantly greater proportion of time in light PA than men. Men grouped in low OA had a significantly greater proportion of time spent in lifestyle PA than women in the same OA grouping. There were no differences in the proportion of time spent in SB or wear time between men and women in any OA grouping. Sensitivity analyses showed significant differences in activity counts/hour, and all activity intensity levels between OA groupings during traditional working hours (9 am–5 pm). The high OA grouping accumulated more activity counts/minute, MVPA, lifestyle, and light PA, and less SB, followed by intermediate and low OA occupations. There were no differences in accelerometer-derived PA and SB variables between OA groupings after work (5–10 pm).

**Table 10:** Accelerometer-derived variable comparison between men and women within OA groupings ^a^: NHANES 2003–2004

	n	TAC M (SE)	Activity counts/min M (SE)	MVPA percentage M (SE)	Lifestyle percentage M (SE)	Light percentage M (SE)	Sedentary percentage M (SE)	Wear time M (SE)
High OA men	252	410,694 (10,303)	465.6 (11.0)	5.1 (0.2)	15.6 (0.4)	32.6 (0.3)	46.7 (0.5)	891.5 (9.4)
High OA women	37	337,066 (32,206)	388.1 (39.2)	3.2 (0.7)	13.4 (1.6)	35.4 (1.8)	48.0 (2.8)	876.2 (16.0)
*p*-value		**0.004**	**0.005**	**<0.001**	0.033	0.118	0.371	0.75
Intermediate OA men	175	346,091 (8743)	390.6 (10.7)	3.7 (0.2)	13.3 (0.5)	31.1 (0.5)	51.9 (0.8)	897.5 (9.5)
Intermediate OA women	125	279,822 (10,002)	326.7 (11.1)	2.1 (0.2)	11.8 (0.5)	34.5 (0.8)	51.4 (1.2)	863.9 (7.6)
*p*-value		**<0.001**	**<0.001**	**<0.001**	0.092	**<0.001**	0.554	0.018
Low OA men	207	317,172 (9801)	359.4 (9.3)	3.9 (0.2)	10.5 (0.5)	26.3 (0.8)	59.2 (1.1)	890.4 (8.0)
Low OA women	316	258,408 (4913)	295.8 (5.0)	2.3 (0.1)	9.1 (0.2)	30.6 (0.2)	57.9 (0.4)	880.4 (4.8)
*p*-value		**<0.001**	**<0.001**	**<0.001**	**<0.001**	**<0.001**	0.227	0.373

^a^Adjusted for age, BMI, race/ethnicity, household income, education, marital status, smoking category, wear time. TAC, total activity counts; Activity counts/min, activity counts per minute; MVPA percentage, proportion of time spent in moderate-to-vigorous physical activity (≥2020 counts) per daily minutes of wear time; Lifestyle percentage, proportion of time spent in lifestyle intensity physical activity (760–2019 counts) per daily minutes of wear time; Light percentage, proportion of time spent in light intensity physical activity (100–759 counts) per daily minutes of wear time; Sedentary percentage, proportion of time spent in sedentary intensity activity (0–99 counts) per daily minutes of wear time; wear time, daily minutes of accelerometer wear time, *p*-value, used alpha = 0.05/7

### OA groupings compared to age-matched, sex-specific population-referenced TAC percentiles

**Fig. 1:**
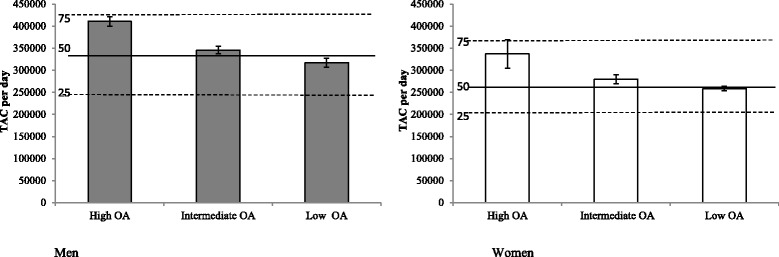
TAC of men and women by OA category against population-referenced TAC percentiles [21]. *TAC* total activity counts per day, *High OA* high occupational activity, *Intermediate OA* intermediate occupational activity, *Low OA* low occupational activity. TAC percentiles (25th, 50th and 75th) for men and women 40 years of age determined from NHANES 2003–2006 [21]

When the PA levels of our sample were compared with a larger sample of U.S. men and women of similar age, men with low OA accumulated TAC slightly below the 50th percentile, while women in low OA had TAC values similar to the 50th percentile (Fig. 1). Men and women in intermediate OA occupations accumulated TAC slightly above the 50th percentile, and men and women in high OA occupations had TAC values above the 50th percentile and close to the 75th percentile compared to sex-and age-matched TAC of U.S. adults.

## Discussion

This study is the first to report observed accelerometer-derived PA and SB variables for a wide variety of occupational categories in U.S. adults. Accelerometry provides an objective measure of movement in everyday life and thus should yield a more valid and reproducible ranking of OA than that achieved earlier by researchers’ best estimates [32]. The information in the paper provides future researchers with detailed measures of the volume and intensity of PA and SB variables that can be compared across the 40 occupational categories based on the 1980 U.S. Census Bureau Classification Coding System. These data also highlight the importance of occupation as a determinant of daily PA and SB.

Previously, occupational categories with indeterminate levels of PA and/or those known to have high variability in PA requirements were considered to be unclassifiable in terms of OA [32]. In fact, more than half (23 of 40) of the occupational categories within NHANES 2003–2004 were considered too ambiguous to classify based on the occupation descriptions of the U.S. Department of Labor [32]. For example firefighters, a specific occupation within the ‘protective services’ category, may be very active when fighting a fire, but the majority of their time may be spent sedentary or in light intensity PA while waiting for a call. Despite the high levels of PA required to be an athlete, they were included in the occupational category ‘writers, artists, entertainers, and athletes’ which was previously categorized as “unclassifiable” due to the assumed lower levels of PA of the other occupations in the category. In our new grouping ‘writers, artists, entertainers, and athletes’ belongs to the low OA grouping, which would suggest that overall number of professional athletes sampled was low. These examples highlight some limitations of relying on job classification as an index of PA [43, 44]. Due to the uncertainty of PA levels of many occupational categories, researchers have predominantly limited their analyses to the occupational categories assumed to have more consistently very high or low levels of OA [12, 21, 45]. The ability to quantify the PA and SB of individuals working in a diverse spectrum of occupational categories with accelerometer data allows for more data-driven grouping into low, intermediate, and high OA. While our tertile approach is arbitrary, it logically splits the occupational categories into three equivalent OA groupings, and avoids having the majority of occupations being considered unclassifiable. Accounting for the variation in PA within each occupational category is still a challenge to overcome, however these data-driven OA groupings may enhance researchers ability to rely on occupational categories as an index of PA, and improve our capability to identify relationships between OA, daily PA and health [46].

In the past relying on occupational categories as a proxy for OA has be useful when gathering information on daily PA of various occupations that involve large amounts of sedentary time or greater amounts of physical labor [32, 46]. The use of objectively measured PA and SB provides data to support the use of occupational categories as a proxy for OA differences. Our study corroborates the utility of the original high and low OA groupings established by King et al. [32]. Only three of the 17 occupational categories previously classified as having high, or low OA were differently categorized using objectively measured PA and SB. ‘Laborers, except construction’ went from high to intermediate OA, ‘material recording, scheduling, and distributing clerks’ went from low to intermediate OA, and ‘motor vehicle operators’ went from low to high OA.

Due to the variety of occupations within some occupational categories PA levels may be quite heterogeneous within an occupational category. Also there is no information regarding the breakdown of all the occupations within each of the 40 occupational categories (i.e., there may have been only one athlete in the ‘writers, artists, entertainers, and athletes’ category). Even within the same occupation variability of PA can be large. For example, cross-country truck drivers spend considerably greater amounts of time sitting compared to local delivery truck drivers who spend a significant amount of time unloading [43]. Additionally, this analysis was not restricted to actual working hours due to the inability to isolate work from non-work time using the data as it was originally acquired. Information regarding regular working hours, shift work or alternative work schedules was not available. Because we were unable to account for variability in occupations within occupational categories, and unable to stratify PA and SB taking place during work or leisure time these limitations could result in non-differential exposure misclassification [47], and limits the ability to accurately interpret these data and ultimately make recommendations about changing behaviors within specific domains.

This study provides benchmark values for accelerometer-derived PA and SB variables, organized by occupation category, which will facilitate comparisons between and across studies using the same classification scheme. Creating tertiles of the summary score from all six accelerometer-derived variables to objectively classify the 40 occupational categories into low OA, intermediate OA, and high OA groupings does have limitations. In some cases, solely focusing on the summary score obscured interesting differences only apparent when closely considering the concurrent rankings of all accelerometer-derived variables between all occupational categories. For example, and consistent with previous research, some low OA occupational categories (e.g., ‘engineers, architects, and scientists’, ‘technicians and related support occupations’, ‘management related occupations’, ‘writers, artists, entertainers, and athletes’, and ‘executives, administrators, and managers’) associated with higher education (>80 % had more than a high school education) and higher income (>70 % earned more than 45K/ year) accumulated relatively higher amounts of MVPA, likely due to increased engagement in leisure time exercise [24, 25, 48]. The higher amount of MVPA relative to the lower summary score in the ‘protective service occupations’ (e.g., firefighters, police and sheriff’s patrol officers, fish and game wardens) was likely due to a combination of increased time spent in vigorous leisure time PA and OA [49]. Because we were unable to separate LTPA from OA, such implications are purely speculative. Although the tertile approach is data-driven, it was possible for two occupations with very similar summary scores to end up in separate OA groupings, for example, ‘motor vehicle operators’ with a summary score of 87 in the high OA group, and ‘supervisors and proprietors, sales occupations’ with a summary score of 89 in the intermediate OA group. Because there was no clear break in the categories, we made an arbitrary split between the 14th and 15th summary scores, despite the small difference in summary score.

In line with previous research, we showed that men were more active (inferred from higher values for TAC, activity counts/minute, and MVPA) than women [37, 40, 50, 51] within the same OA groupings after adjusting for characteristics known to be related to PA. In addition our data highlighted differences in PA of men and women within the same occupational category, and showed obvious differences in the proportion of men and women employed in specific occupational categories. For example, in our sample, only men were employed in many high OA occupational categories, while several low OA occupational categories were 100 % women. Due to gender differences in PA, the proportion of women in an occupational category may affect the results. The proportion of men and women employed as ‘engineers, architects, and scientists’, ‘technicians and related support occupations’, ‘management related occupations’, ‘executives, administrators, and managers’, protective services’, and ‘writers, artists, entertainers, and athletes’ may be another explanation for why these low OA occupational categories had higher than expected MVPA relative to their summary score. These six low OA occupational categories contained 60 % men, while the remaining eight low OA occupational categories comprised only 25 % men.

A number of additional analytical limitations to this analysis must be acknowledged. Operating under the assumption that OA is a major determinant of PA for many adults, the sample was restricted to adults working full-time, therefore the results may not be generalizable to populations employed part-time. Because of numerous exclusion criteria a large proportion of the adult population was excluded from the analysis, therefore the study population may not be entirely representative of the U.S. adult population. Of the 40 occupational categories, 16 were represented by less than 20 individuals. Therefore, the expected accelerometer-derived variables for some occupational categories should be interpreted with caution as they may not reflect national estimates of workers in these categories. We considered only reporting the accelerometer-derived variables for the 24 categories with a sample size greater than 20, as recommended by the NCHS [52]. However, despite these relatively small sample sizes, the majority of accelerometer-derived variables displayed a relative standard error (the standard error as a percent of the measure itself, much like the coefficient of variation) within the range deemed acceptable (<30 %) by the NCHS [42]. Thus, we opted for a more comprehensive presentation of all 40 occupational categories. We clearly indicated when violations to the relative standard error rule occurred and presented the sample size of each occupational category within the tables. Because of sample size limitations we chose to present a more conservative descriptive epidemiology of accelerometer-derived variables rather than conducting statistical comparisons between occupational categories, and men and women. However, showing the number of men and women in each category of the current sample was informative, especially in cases where the whole category was men, or exclusively women. As the number of women entering the work force has grown in the last half century, it would be useful to develop sex-specific estimates for OA in different occupational categories. Unfortunately, the occupational categories for NHANES 2005–2006 were different, so enlargement of the sample by incorporation of multiple waves of NHANES was not possible.

A major strength of this study was the use of accelerometers to objectively measure PA. At the time of data collection, uniaxial accelerometers were the method of choice, and weaknesses related to uniaxial, count-based PA measurement must be acknowledged. Accelerometers do not capture all types of PA, nor do they provide information on the type of PA performed, and their ability to accurately identify time spent in specific intensity categories has been questioned [53–55]. When worn at the waist accelerometers are most sensitive to ambulatory PA, and therefore the additional intensity of carrying loads, or other upper body movements is not captured [37]. This instrument has been validated against measured activity energy expenditure [56, 57], however it has not been validated for capturing the PA patterns characterizing OA in different occupations. The PA patterns characterizing OA (e.g., mostly sitting, and standing, with little walking, engaging in repetitive tasks) differ significantly from LTPA patterns which usually involve dynamic movements that engage large muscle groups resulting in increased whole-body metabolism and cardiac output, and are often emphasized for their the health-promoting capacity [16]. Considering the diverse movement requirements of different occupations (e.g., degree of static work, upper body work, standing, moving, lifting and loading occupations) [16], the observed differences in PA between the occupational categories may be more a reflection of the ambulatory movement captured by the uniaxial waist worn accelerometer than differences in other forms of OA. If carrying loads and upper body movements (e.g., food trays, pitchers/pots for ‘waiters and waitresses’ who had the largest proportion of light PA) were considered, the PA intensity may have been considerably higher.

The movement captured by the accelerometer reflects the accumulation of PA at home, in transit, during leisure time, in addition to time at work [37]. The differences in accelerometer-derived PA and SB variables between OA groupings occurred during traditional work hours (9 am–5 pm). Outside of traditional work hours OA groupings had similar levels of PA and SB. This supports our analytical assumption that the majority of workers were working a traditional day shift, and that the differences in accelerometer-derived PA and SB variables were likely due to OA. However, we must acknowledge our inability to separate PA and SB taking place during work and leisure time, and previous research which indicates that OA and LTPA is profoundly different in varying occupations. Blue collar workers with physically strenuous occupations and frequent overtime work are significantly less likely to engage in LTPA, while white collar workers may engage in greater LTPA, after a sedentary day at work [24, 25, 48].

An additional concern regarding waist worn, uniaxial accelerometers is their inability to accurately differentiate between sitting and standing postures [58–60], which can result in the misclassification of standing-light work, a light intensity PA, as sedentary [61–63]. Some occupations traditionally spend much of the work day in an upright posture, standing and/or moving around in light intensity behaviors (e.g., teachers, cooks, retail, waiters and waitresses). The increased energy expenditure and postural demands of standing compared with sitting may be an important distinction to consider when evaluating health outcomes [60, 64]. For assessing and differentiating between sitting and standing postures between different occupations, a thigh-mounted accelerometer like the ActivPAL monitor [65] or Actigraph [66], or triaxial accelerometer (ActiGraph GT3X+) at the thigh or hip may provide greater accuracy [67].

## Conclusions

Objectively measured PA allowed for a more precise estimate of the amount of PA and SB associated with different occupational categories, and made it possible to systematically classify 40 different occupational categories into three distinct OA groupings. An individual’s occupational category appeared to have a great influence on daily PA and SB. This information provides new opportunities to explore the relationship between OA and health outcomes. It is also important for the design and implementation of programs and policies to improve health, productivity, and reduce work related injury of the employed population. Future occupational epidemiological research is needed to understand how variations in OA, LTPA, transportation, and household PA interact to influence engagement in beneficial amounts of daily PA, and influence health [17]. In addition, future research should continue to refine recommendations of maximal levels of OA, because strenuous OA can have health-detrimental impacts such as musculoskeletal disorders, decreased work ability and absence due to work related sickness or injury [68]. The results reported here highlight the scarcity of the data available for certain occupational categories to conduct and inform such studies. A larger sampling of underrepresented occupational categories, and a wide range of unique occupational categories would benefit future research concerned with the impact of occupation on PA and SB. The observed values presented in this paper are an important resource that should be expanded and refined as future changes occur in OA and as occupational categorization systems evolve.

## Abbreviations

OA: Occupational activity
PA: Physical activity
SB: Sedentary behavior
NHANES: National Health and Nutrition Examination Survey
TAC: Total activity counts/day
MVPA: Moderate-to-vigorous physical activity
NCHS: National Center for Health Statistics
SE: Standard error
